# Genetic Variants of Obesity in Malaysia: A Scoping Review

**DOI:** 10.3390/genes15101334

**Published:** 2024-10-17

**Authors:** Siti Sarah Hamzah, Liyana Ahmad Zamri, Norhashimah Abu Seman, Nur Azlin Zainal Abidin

**Affiliations:** Endocrine and Metabolic Unit, Nutrition, Metabolic & Cardiovascular Research Centre, Institute for Medical Research, National Institutes of Health, Ministry of Health Malaysia, Setia Alam 40170, Selangor, Malaysia; liyana.az@moh.gov.my (L.A.Z.); nor.hashimah@moh.gov.my (N.A.S.); nurazlin.za@moh.gov.my (N.A.Z.A.)

**Keywords:** obesity, biomarkers, Malaysia, scoping review

## Abstract

Background: Obesity is a pressing public health issue in Malaysia, involving not only excess weight but also complex metabolic and physiological changes. Addressing these complexities requires comprehensive strategies, including understanding the population-level differences in obesity susceptibility. This review aims to compile the genetic variants studied among Malaysians and emphasize their implications for obesity risk. Methods: Relevant articles published up to March 2024 were extracted from the Scopus, PubMed, and ScienceDirect databases. The review process was conducted in accordance with the PRISMA-ScR guidelines. From an initial pool of 579 articles, 35 of these were selected for the final review. Results: The identified gene variants, including *LEPR* (K656N), *LEP* (G2548A—Indian only), *ADIPOQ* (rs17366568), *UCP2* (45bp-I/D), *ADRB3* (rs4994), *MC3R* (rs3827103), *PPARγ* (pro12Ala—Malay only), *IL1RA* (intron 2 VNTR), *NFKB1* (rs28362491), and *FADS1* (rs174547—Indian only), showed significant associations with obesity as measured by the respective studies. Conclusions: Overall, more intensive genetic research is needed, starting with population-based profiling of genetic data on obesity, including among children. Sociocultural contexts and environmental factors influence variations in genetic elements, highlighting the need for targeted interventions to mitigate the impacts of obesity in the population.

## 1. Introduction

The World Health Organization (WHO) identifies overweight and obesity as the fifth leading risk for global mortality, with elevated BMI contributing to diseases like cardiovascular diseases, diabetes, and certain types of cancer [1,2]. Obesity, once considered a problem predominantly in high-income countries, has now become an increasing public health issue worldwide.

In Malaysia, the trends in overweight, obesity, and abdominal obesity have continued to rise, as evidenced by the National Health and Morbidity Survey (NHMS) in 2011 (29.4%, 15.1%, and 45.4%) and 2015 (30%, 17.7%, and 48.6%) [3,4]. Currently, obesity remains a major health challenge in the country, with no sign of abating. The latest NHMS report reveals that over half of the adult population is affected by weight issues, with 54.4% being overweight or obese and 54.5% having abdominal obesity [5].

Recently, the Health Research Priorities for the 12th Malaysia Plan (12MP-HRP) 2021–2025 have identified 15 priority research areas related to overweight and obesity, with one of them being the need to investigate the role of genetic factors in the development of overweight and obesity [6]. This shows that studies in this area and scientific data on this topic are still scarce in Malaysia.

Obesity is widely recognized as a polygenetic disease influenced by interactions between genes and the environment, including factors such as diet, lack of physical exercise, microbiome, and chemical contaminants, all of which can alter gene expression [7]. In addition to environmental, social, and economic factors, phenotypic heterogeneity exists among overweight and obese individuals due to interactions at the molecular, genetic, and cellular levels [8]. Another form of obesity also exists, known as monogenic obesity, in which mutations in specific genes cause early-onset of obesity, often from infancy or early childhood. Recently, gene discovery studies have found common fundamental biology between polygenic and monogenic obesity, with central nervous system (CNS) and neuronal pathways that control food intake being major drivers of body weight in both forms [9]. Early evidence also shows that individuals’ polygenic susceptibility to obesity may partly influence the expression of mutations that cause monogenic obesity [10]. Therefore, it is crucial to identify population-based genetic variants associated with obesity to enhance understanding of their predisposition for improved diagnosis and management.

Given these considerations, this scoping review aims to compile and present genetic research on obesity conducted in Malaysia up to March 2024. To date, no review has specifically focused on this aspect within Malaysia. This review consolidates the studied genetic variants and their associations with obesity and offers recommendations for future research based on the limitations of current studies. Ultimately, this review seeks to effectively guide future obesity research in Malaysia and address the gaps highlighted in the 12th Malaysia Plan.

## 2. Methods

### 2.1. Protocol

The framework for analyzing this scoping review was conducted following the methodology outlined by Arksey and O’Malley [11]. The scoping review protocol was prospectively registered with the Open Science Framework (https://osf.io/qyzka, accessed on 11 July 2024). Guidelines for conducting a scoping review were adhered to [12], along with the PRISMA-ScR (Preferred Reporting Items for Systematic Review and Meta-analyses extension for Scoping Reviews) guidelines for reporting [13].

### 2.2. Identification of Relevant Studies

A systematic literature search was performed using a predefined search strategy across Scopus, PubMed, and ScienceDirect databases. This search was conducted by at least two independent researchers. The search included articles published from the earliest available date to March 2024, as research on this topic was limited in Malaysia. English language terms were used and review articles including scoping, systematic, narrative, meta-synthesis, and meta-analysis were omitted. Boolean operators (AND, OR) were utilized to combine words. The identified records were exported to EndNote and independently screened for inclusion by four reviewers. Disagreements were addressed through discussion until a consensus was achieved.

### 2.3. Inclusion Criteria

The inclusion criteria were as follows:

- Peer-reviewed articles, including original research and clinical studies;
- Human-based research;
- Study conducted among Malaysian (Malay, Chinese, Indian, indigenous people, bumiputra Sabah and Sarawak);
- English and Malay language;
- Any articles published until March 2024.

The exclusion criteria were as follows:

- Books, book chapters, and book reviews;
- Review articles (systematic, meta-analysis, meta-synthesis, scoping, narrative);
- Animal studies;
- Non-Malaysian population;
- Perspective, opinion, and commentary in peer-reviewed journal;
- Non-genetics or obesity studies.

### 2.4. Data Extraction

Data extraction from the included studies was conducted using a standardized data extraction sheet in Microsoft Excel 2021 (Microsoft Corporation, Redmond, WA, USA). The extraction process included recording the year of publication, scientific study title, authors, objectives, sex distribution, sample size, ethnicity, comparison group details, and findings.

## 3. Results

### 3.1. Study Characteristics

**Figure 1:**
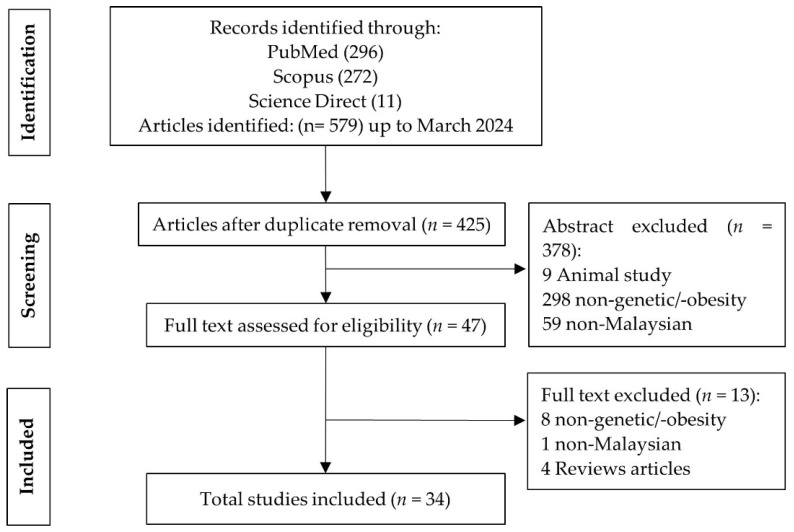
PRISMA flowchart outlining selection process for including studies in the review.

Based on the keyword searches, 579 potentially eligible records were identified, out of which 153 articles were excluded due to redundancy. Following abstract screening, 378 articles were further excluded, leaving 47 articles for full-text screening. Subsequently, 13 studies were excluded during the full-text review, resulting in the inclusion of 34 studies for data extraction and analysis. In total, 391 articles were removed during screening for following reasons: 9 studies involved animal subjects, 306 were unrelated to genetics or obesity, 60 articles were conducted among non-Malaysians and 16 were review papers (Figure 1).

The characteristics of the included studies are summarized in Table 1. The first genetic study on obesity in Malaysia was published in 2009 by Liew et al. followed by an increasing trend in research on gene polymorphisms and their association with obesity starting from 2018 until the present. Most studies focused on university students and local community members attending health clinics, with objectives primarily centered around the relationship between specific gene variants and obesity or health parameters associated with obesity. Only three studies were conducted among children. The majority of studies involved three or more ethnic groups (*n* = 19), while others focused exclusively on Malay participants or had more than 75% Malay participants (*n* = 11), Chinese participants (*n* = 2), or combined Chinese and Indian participants (*n* = 2). However, 99% of the studies did not stratify outcomes by ethnic group (except for the study by Kok et al.) but rather compared obese and non-obese individuals. It is noteworthy that all included studies had relatively small sample sizes, ranging from 150 to 1200 participants, which may impact the validity and generalizability of their research findings.

### 3.2. Genetic Variants of Interest and Risk of Obesity

Forty-two variants of genes directly or indirectly involved in susceptibility to obesity were identified (Table 2). Each study examined the association between these gene variants and obesity, typically reporting results in terms of Minor Allele Frequency (MAF) and/or Odds Ratio (OR). Among these, the genetic variants of *LEPR* (K656N), *LEP* (G2548A- Indian only), *ADIPOQ* (rs17366568), *UCP2* (45bp-I/D), *ADRB3* (rs4994), *MC3R* (Rs3827103), *PPARγ* (pro12Ala—Malay only), *IL1RA* (intron 2 VNTR), *NFKB1* (rs28362491), and *FADS1* (rs174547—Indian only) have been significantly associated with obesity risk in Malaysians.

However, no significant association with obesity was reported for the genetic variants of *LEP* (A19G), *LEPR* (K109R, Q223R), *PYY* (R72T), *NPY* (rs16147T, rs161139C), *PPAR* (L162V), *PPAR2* (C161T), *PPARδ* (T294C), *UCP1* (-3826 A/G), *UCP3* (-55C/T), *CARTPT* (rs2239670), POMC (Rsal), *MC4R* (V1031), *FTO* (rs9930506, rs9939609, rs17817288, rs9930501, rs9932754), *ADIPOQ* (rs3774261), *INSIG2* (rs7566605), *RETN*, *DRD2* (Taq1A, Taq1B, Taq1C), *VDR* (bsml), *IRX* (rs3751723), *FASN* (rs4246445, rs2229425, rs2228305,rs2229422), and *ADRB2* (rs1042713).

### 3.3. Protein–Protein Interaction (PPI) Network

**Figure 2:**
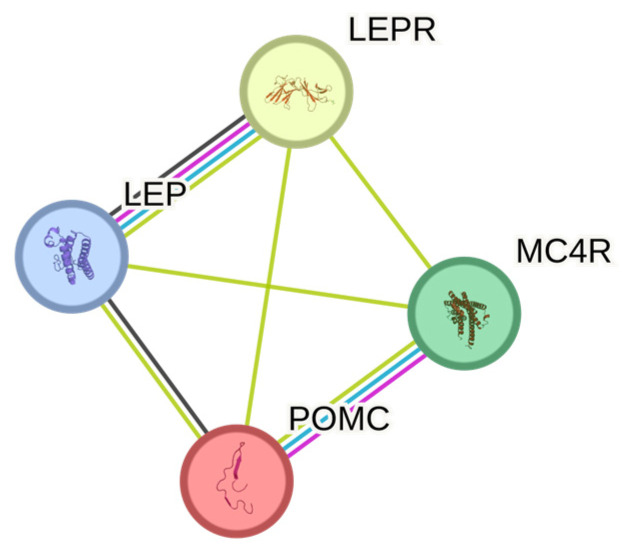
STRING protein networks of genes associated with monogenic obesity.

Out of the thirty-three genes for which selected genetic variants were reported as significantly associated with obesity, only four genes (*LEP, LEPR, POMC*, and *MC4R*) are known to be linked with monogenic obesity. Mutations in these genes can cause early-onset obesity which is not easily influenced by environmental factors alone, unlike polygenic obesity. Since the majority of the reviewed articles are highly heterogenous, biased in terms of selecting candidate genes, and the studies were mostly underpowered, only the genes associated with monogenic obesity were selected for protein—protein interaction analysis. The STRING database shows that interactions between *LEP, LEPR, POMC*, and *MC4R* have been reported from various experimental data, curated databases, and co-expression studies (Figure 2). Nodes in these networks represent proteins, and the edges denote their interactions. The roles of these genes in many biological processes and important molecular pathways are also well established. Examples of Gene Ontology (GO) biological processes involving some or all of the genes include response to a melanocyte-stimulating hormone, leptin-mediated signaling pathway, regulation of appetite, and regulation of feeding behavior. In terms of KEGG pathways, examples include adipocytokine signaling pathway and AMPK signaling pathway (Table 3).

## 4. Discussion

Review findings suggest that there was a growing focus on genetic variants associated with obesity in Malaysians. This trend may be attributed to substantial evidence indicating that specific genetic variants may affect populations differently, influenced by factors such as sociocultural contexts, dietary habits, and patterns of body fat distribution [48]. It was noted that the majority of the included studies (94%) were conducted among adults in universities and local health clinics. However, defining early predictors of obesity is crucial, as the National Health Morbidity Survey 2022 (NHMS 2022) reported that childhood obesity is currently a major health problem in Malaysia, with one in three teens aged 13–17 being overweight or obese [49]. Therefore, while it is more convenient to recruit adults, there is also a need for more biomarker studies involving children and adolescents. This would simultaneously address the scarcity of scientific data on the genetics of childhood obesity, as identified in the Health Research Priorities for the 12th Malaysia Plan (12MP-HRP) [6].

The present review also found that a considerable number of genetic variants have been explored. Out of 42 gene polymorphisms investigated, fewer than half were reported to have a significant association with obesity by the authors, and several studies investigated overlapping targets. For example, variants like *LEPR* Q223R [14,23,25,46] *LEP* Q2548A [23,25,37,46], *LEP* A19G [14,37], *PPAR* L162V and *PPAR2* C161T [24,38], *FTO* rs9930506 [38,45], *UCP2* 45-bp I/D [15,27], *DRD2* Taq1A, Taq1B and Taq1D [39,44]. Notably, the findings across these studies were consistent, showing that none of the genetic variants were linked to obesity, except for *LEP* G2548A [23], which may be associated with overweight/obesity among Indian males; *UCP2* 45 bp I/D [27] with overall adiposity among Malaysian women; and *DRD2* polymorphisms with eating behavior but not with obesity [39,44]. It is worth noting that these findings may, in part, reflect the small sample sizes used in the studies and the fact that the claimed significance level may not be entirely reliable due to the insufficient statistical power. To address this issue more effectively, studies with larger sample sizes and adequate power are needed to produce more reliable and valid findings that contribute more effectively to scientific knowledge.

Furthermore, this review has identified contradictory results regarding the association of well-established *FTO* gene variants with obesity risk among Malays, Chinese, and Indians. A research group in Singapore found that *FTO* variants, especially rs9939609, which are common in European populations, were significantly associated with obesity in Chinese and Malays but not in Asian Indians [50]. However, there was no evidence for this SNP or other *FTO* regions in obesity and obesity-related parameters in either Chinese, Malays, or Indians in Malaysia [21]. Nevertheless, these results further emphasize the variation in genetic elements among individuals that influence susceptibility to obesity. This is supported by Karra et al., who showed that people with two high-obesity-risk *FTO* variants have a 70% increased risk of becoming obese compared to those with low-obesity-risk variants [51]. Therefore, given that the targets examined in the included studies were based on evidence from various populations, it is essential for researchers in Malaysia to first conduct gene profiling among obese individuals. This preliminary step will help to identify specific biomarker targets for obesity, paving the way for more focused genetic investigations.

Due to the lack of homogeneity in the samples from most of the reviewed articles, only the protein interactions between studied genes related to monogenic obesity are analyzed using STRING. Monogenic obesity is relatively rare, follows a Mendelian pattern of inheritance, and is usually characterized by severe obesity, unlike the polygenic form of obesity, which often arises from the cumulative effect of multiple genetic variants, each contributing a small effect on obesity risk, as well as environmental factors. Genes such as *LEP, LEPR, POMC,* and *MC4R* are known to play crucial roles in various important biological processes and pathways. The most significant shared pathway relates to the adipocytokine signaling pathway. This pathway involves cytokines and adipokines produced by adipocytes and is interconnected with other pathways such as the AMPK signaling pathway, regulating processes such as the metabolism, inflammation, and energy balance. Some components of adipokines, including leptin, adiponectin, resistin, Interleukin-6 (IL-6), and Tumor Necrosis Factor-α (TNF-α), have been associated with the development of insulin resistance, obesity, and related health conditions [52,53,54,55]. Understanding the characteristics of genetic variants that cause each form of obesity is crucial for more precise disease management.

This scoping review has several limitations. Firstly, most of the included studies had small sample sizes and suboptimal study designs. Secondly, the majority of study participants were drawn from institutional and healthcare settings, which may limit the generalizability of the results. Thirdly, the studies encompassed a wide range of ages, and these variations should be considered when interpreting the results. Finally, the bibliographic search was conducted using only three databases, which might have led to the omission of some relevant articles.

For future obesity genetic research, it is crucial for researchers to adopt appropriate study designs and ensure the inclusion of larger sample sizes to strengthen the validity of the findings. Additionally, it is important to include participants from community settings to ensure the findings are applicable to a broader population. Given the significant role of gene–environment interactions in the onset of obesity, large-scale epigenetic studies are needed to identify novel genetic variants specific to the Malaysian population. Leveraging advanced technologies, such as long-read sequencing, can help overcome the limitations of genome-wide association studies (GWAS), which primarily capture common genetic variants.

## 5. Conclusions

In summary, genetic biomarker research on obesity among Malaysians remains limited in scope, primarily focusing on well-known genes or gene variants. While targeting established gene variants can be one research strategy, it is more compelling to establish comprehensive biomarker genetic profiles related to obesity across different age groups—from children to adults—in the local population.

## Figures and Tables

**Table 1:** Participants characteristics.

No	Study (Author/Year)	Objectives	Sample Size	Participants characteristics (Gender/Ethnicity/Obesity Status)
1	Liew et al., 2009 [14]	(i) To investigate the prevalence of the leptin gene *(LEP*) A19G and leptin receptor gene (*LEPR*) K109R, Q223R, and K656N variants and their possible association with obesity. (ii) To investigate the prevalence of associated obesity risk factors in Malaysian university students of Setapak, Kuala Lumpur.	200	Males: 85, Females: 115/mean age: 21.22 ± 2.85 years/Malay: 3 (2.1%); Chinese: 96 (67.1%); Indian: 36 (25.2%); Others: 3 (2.1%)/Non-obese: 143; Obese: 57
2	Yiew et al., 2010 [15]	To examine the prevalence of these gene polymorphisms and their possible potential link to obesity among university students.	256	Males: 140, Females: 116/mean age 21.7 ± 1.7 years/Chinese: 240 (93.8%)/Non-obese: 170; Overweight: 67; Obese: 19
3	Chan et al., 2011 [16]	To examine the association between the R72T variant of the Peptide Tyrosine-Tyrosine (*PYY*) gene and obesity, along with related anthropometric measurements, among the cohort from Kampar Health Clinic in Malaysia.	197	Males: 78, Females: 119/mean age: 55.1 ± 11.0 years/Malay: 75 (38.1%); Chinese: 77 (39.1%); Indian: 40 (20.3%); Others: 5 (2.5%)/Non-obese: 98; Obese: 99
4	Lisa et al.,2011 [17]	To examine the association between the CART prepropeptide gene (*CARTPT*) rs2239670 variant and obesity, along with related anthropometric indicators, among patients at a health clinic in Kampar, Perak, Malaysia.	300	Males: 115, Females: 185/Malay/Peninsular Bumiputera: 98 (32.7%); Chinese: 141 (47.0%); Indian: 61 (20.3%)/Non-obese: 163 (mean age: 53.18 ± 15.85 years); Obese: 137 (51.46 ± 12.40)
5	Lee et al., 2012 [18]	To explore the prevalence of the RsaI SNP in the 5′-untranslated region (UTR) of the *POMC* gene and its potential association with obesity.	302	Males: 120, Females: 182/Malay: 92; Chinese: 141; Indian: 64; Others: 5/Non-obese: 160; Obese: 142
6	Chua et al., 2012 [19]	To assess the prevalence of the Melanocortin receptor 4 (*MC4R*) V103I gene variant and its relationship with obesity among a group of patients attending the Kampar Health Clinic.	254	Males: 101; Females: 153/mean age: 52.27 ± 14.2 years/Malay: 74 (29.1%); Chinese: 124 (20.1%); Indian: 51 (20.1%); Others: 5 (2.0%)/Non-obese: 136; Obese: 118
7	Apalasamy et al., 2012 [20]	To examine whether SNPs and linkage disequilibrium (LD) blocks in different regions of the *FTO* gene are associated with obesity susceptibility in Malaysian Malays.	587	Malay: 587 (100%)/mean age: 48.29 ± 9.89 years/Non-obese: 429; Obese: 158
8	Chey et al., 2013 [21]	To examine the association between the *FTO* rs9939609 variant and obesity in a multi-ethnic Malaysian population.	324	Males: 126; Females: 198/age: 21 to 80 years old/Malay: 98; Chinese: 158; Indian: 68/Non-obese: 178; Obese: 146
9	Apalasamy et al., 2013 [22]	To genotype *MC4R* gene variants and evaluate the genetic link between *MC4R* SNPs and obesity-associated parameters.	652	Males: 293; Females: 359/Malay: 483 (100%)/Non-obese: 483; Obese: 169
10	Ng et al., 2014 [23]	To assess the prevalence of overweight and obesity among Malaysian adolescents and investigate the association of specific polymorphisms with overweight, obesity, or excess body fat in this group.	613	Males: 248 (40.5%); Females: 365 (59.5%)/mean age: 14.8 ± 1.3 years; Malay: 241; Chinese: 219; Indian: 153/Non-obese: 470; Overweight/Obese: 143
11	Apalasamy et al., 2014 [24]	To examine the association between the *ADIPOQ* rs17366568 and rs3774261 SNPs with obesity, as well as their association with adiponectin levels, in Malaysian Malays.	574	Malay: 574 (100%)/Non-obese: 424 (mean age: 46.17 ± 5.32 years); Obese: 150 (mean age: 45.61 ± 7.37 years)
12	Fan and Say, 2014 [25]	To examine the prevalence of SNPs in the leptin gene (*LEP*) [A19G and G2548A] and the leptin receptor gene (*LEPR*) [K109R and Q223R], and their associations with fasting plasma leptin levels and obesity in a suburban population in Kampar, Perak.	408	Male: 169; Female: 239/mean age: 52.4 ± 13.7 years/Malay: 148; Chinese: 177; Indian: 83/Non-obese: 218; Obese: 190
13	Apalasamy et al., 2014 [26]	To investigate the association between the rs7566605 SNP and obesity, as well as other metabolic parameters, in Malaysian Malays.	672	Malay: 672 (100%)/Non-obese: 500 (mean age: 46.47 ± 7.06 years); Obese: 172 (mean age: 47.97 ± 6.05 years)
14	Say et al., 2014 [27]	To assess the prevalence of the *UCP2* 45-bp I/D polymorphism and its potential association with obesity (measured by BMI), overall adiposity (measured by total body fat percentage), and central adiposity (measured by waist-to-hip ratio) in a representative sample of the multi-ethnic Malaysian population.	926	Males: 416, Females: 510/Malay: 102; Chinese: 672; Indian: 152/Non-obese: 661; Obese: 265
15	Apalasamy et al., 2015 [28]	(i) To examine the association between polymorphisms in the resistin gene and obesity in a homogeneous Malaysian Malay population. (ii) To explore the association between resistin levels and specific SNPs and haplotypes of the *RETN* gene.	631	Malay: 631 (100%)/Non-obese: 469 (mean age: 48.33 years); Obese: 162 (mean age: 48.43 years)
16	Apalasamy et al., 2015 [29]	To examine the association between the rs1042714 SNP and obesity-related parameters.	672	Male: 300; Female: 372/mean age: 48.22 ± 10.05 years/Malay: 672 (100%)
17	Chia et al., 2015 [30]	To investigate the association of peroxisome proliferator-activated receptor (*PPAR*) genes *PPARα* L162V, *PPARγ2* C161T, and *PPARδ* T294C single nucleotide polymorphisms (SNPs) with obesity and metabolic syndrome (MetS) in a multi-ethnic population in Kampar, Malaysia.	307	Males: 124; Females: 183/mean age 53.3 ± 14.2 years/Malay: 97; Chinese: 85; Indian: 55/Non-obese: 127, Obese: 180
18	Zain et al., 2015 [31]	(i) To assess the impact of *NPY* rs5574 and rs16147 variants on the risk of obesity in Asians. (ii) To perform a meta-analysis summarizing the effects of these variants, including the extensively researched rs16139.	942	Males: 264 (28%); Females: 678 (72%)/age: 13 years/Malay: 74%; Chinese: 13%; Indian: 10%; Others: 3%/Non-obese: 680; Overweight/obese: 262
19	Zaharan et al., 2018 [32]	To investigate potential associations between adiposity parameters and selected SNPs among the Malaysian Health and Adolescents Longitudinal Research Team study (MyHeARTs).	1179	Males: 39%; Females: 61%/age: 15 years old/Malay: 79%; Chinese: 7%; Indian: 9%; Others: 5%/Non-obese: 76%; Overweight/Obese: 24%
20	Zahri et al., 2016 [33]	To determine the genotypic and allelic frequencies of the *PPARƔ2* gene and assess its association with lipid profiles, anthropometric measurements, and obesity susceptibility in Malay individuals.	217	Malay: 217 (100%)/Non-obese: 123 (mean age: 33.59 ± 10.54 years); Obese: 94 (mean age: 39.18 ± 9.97 years)
21	Shunmugame et al., 2016 [34]	To investigate the association between the adrenergic receptor α2A (*ADRA2A*) rs553668 and angiotensin-converting enzyme (ACE) I/D SNPs with obesity traits (body mass index—BMI; waist-hip ratio—WHR; total body fat percentage—TBF) in a Malaysian population.	214	Males: 99; Females: 115/mean age: 26.27 ± 11.93 years/Malay: 45; Chinese: 116; Indians: 53/Non-obese: 142; Obese: 72
22	Kok et al., 2017 [35]	To examine the association of *IL1RA* and *IL4* VNTRs with obesity and adiposity in 315 Malaysian individuals.	315	Males: 128; Females: 187/Malay: 23; Chinese: 251; Indian: 41/Non-obese: 261; Obese: 54
23	Rahmadhani et al., 2017 [36]	To investigate the association between BsmI polymorphism and risk of vitamin D deficiency, obesity, and insulin resistance in adolescents residing in a tropical country.	941	Males: 261 (28%); Females: 680 (72%); age: 13 years old/Malay: 702 (75%); Chinese: 121 (13%); Indian: 94 (10%); Others: 24 (2%)/Non-obese: 629 (67%); Overweight: 104 (11%); Obese: 208 (22%)
24	Shamsuddin et al., 2018 [37]	To investigate the association of SNPs and haplotype of the Leptin gene, specifically G2548A, A19G, and H1328080 with obesity in Malays from Terengganu.	249	Malay: 249 (100%)/Non-obese: 101; Overweight: 148; Obese: 54
25	Mitra et al., 2018 [38]	To assess (i) the impact of *FTO* rs9930506 on obesity and related anthropometric and biochemical parameters, and (ii) how diet influences the relationship between *FTO* rs9930506 and obesity phenotypes.	178	Males: 24; Females: 154/Non-obese: 99; Obese: 79
26	Lek et al., 2018 [39]	To examine the association between *DRD2* Taq1A, Taq1B, and Taq1D gene polymorphisms with eating behavior (i.e., the preference, intake frequency, craving of high-fat foods) and obesity.	394	Males: 161 (mean age: 20.9 ± 0.13 years); Females: 233 (mean age: 20.9±0.11 years)/Chinese: 308; Indian: 86/ Non-obese: 327; Obese: 67
27	Chong et al., 2018 [40]	To examine the association between the Iroquois homeobox 3 (*IRX3*) rs3751723 polymorphism and increased risk of obesity in the Malaysian population through a case-control study and a meta-analysis.	1030	Non-obese: 694 (mean age: 25.91 ± 9.75 years); Overweight: 223 (mean age: 26.91 ± 12.98 years); Obese: 113 (mean age: 26.45 ± 11.97 years)
28	Chong et al., 2018 [41]	To explore the association between fatty acid synthase (*FASN*) rs4246445, rs2229425, rs2228305, and rs2229422 SNPs with the risk of overweight and obesity in the Malaysian population.	1030	Males: 620; Females: 410/Non-obese: 694 (mean age: 23.41 ± 9.81 years); Overweight: 223 (mean age: 23.34 ± 9.78 years); Obese: 113 (mean age: 23.45 ± 9.88)
29	Mitra et al., 2019 [42]	To assess (i) the relationship between *ADRB2* rs1042713 and obesity as well as related metabolic parameters, and (ii) the impact of dietary nutrients on these associations in Malaysian adults.	178	Males: 24; Females: 154/Non-obese: 99; Obese: 79
30	Al-Shajrawi et al., 2020 [43]	To investigate the role of variants in *NFKB1* (rs28362491) and *HIF1* (rs11549465) in relation to obesity in Malay individuals.	188	Males: 70; Females: 118/Malay: 188 (100%)/Obese: 93 (37.9 ± 2 9.2 years); Non-obese: 95 (mean age: 32.24 ± 12.1 years)
31	Lim et al., 2020 [44]	To examine how dopamine type 2 receptors (*DRD2*) gene variants (*ANKK1/DRD2* Taq1A, *DRD2* Taq1B, and *DRD2* Taq1D) influence eating behaviors (i.e., cognitive restraint eating (CR), emotional eating (EE), and uncontrolled eating (UE)) and their association with obesity.	394	Males: 125; Females: 269/Malay: 32, Chinese: 329, Indian: 32; Aborigine: 1
32	Tan et al., 2020 [45]	To examine the combined effect of the *FTO* rs9930501, rs9930506, and rs9932754 variants and *ADRB2* rs1042713 and rs1042714 using polygenic risk scores (PRSs) on (1) the odds of obesity and (2) changes in dietary, anthropometric, and cardiometabolic parameters following a high-protein, calorie-restricted, high-vitamin E, high-fiber (Hipcref) diet intervention in Malaysian adults.	Cross sectional:178, RCT 128	Males: 24; Females/Age: ≥18 years: 154/Malay: 86; Chinese: 42; Indians: 50/Non-obese: 99; Obese: 79
33	Mohanraj et al., 2022 [46]	To investigate the association between sleeping habits, eating behavior, stress indicators, and plasma leptin levels, as well as its genomic polymorphisms, among different racial groups within a young adult healthcare student population in Malaysia.	185	Males: 89; Females: 96/Malay: 61; Chinese: 45; Indian: 56; Others: 23/Non-obese: 129; Overweight/Obese: 56
34	Ching et al., 2023 [47]	To assess how the rs174547 variant in the fatty acid desaturase 1 (*FADS1*) gene interacts with macronutrient intakes such as carbohydrates (especially fiber), protein, and fat and its impact on abdominal obesity among middle-aged Malaysian vegetarians of Chinese and Indian ethnicity.	163	Males: 50 (30.7%); Females: 113 (69.3%)/mean age: 50 ± 5 years/Chinese: 95; Indian: 68

**Table 2:** List of studied genetic variants associated with monogenic and polygenic obesity. The Minor Allele Frequency (MAF), Odds Ratio (OR), and findings on the association with obesity for each variant presented were taken directly from the respective studies.

(a) Monogenic Obesity				
No	Author	Gene Variants	Outcomes (Minor Allele Frequency (MAF)/Odd Ratio (OR)	Estimated Power of Study
1	Liew et al., 2009 [14]	Leptin (*LEP*) gene A19G Leptin Receptor (*LEPR*) gene K656N, Q223R, and K109R	**MAF**: *LEP* A19G (0.52) and *LEPR* K109R (0.43), *LEPR* Q223R (0.36), 656N allele (0.31). **OR**: N/A. **Finding of the study**: Only *LEPR* K656N was associated with obesity.	0.545
2	Chua et al., 2012 [19]	Melanocortin receptor 4 (*MC4R*) V103I gene variant	**MAF**: N/A. **OR**: N/A. **Finding of the study**: *MC4R* V103I variant does not show a direct correlation with obesity in the studied cohort.	0.58
3	Lee et al., 2012 [18]	RsaI SNP site in the 5′-untranslated region (UTR)of *POMC*	**MAF**: 0.31. **OR**: N/A **Finding of the study**: RsaI alleles and genotypes were not identified as risk factors for obesity.	0.05
4	Apalasamy et al., 2013 [22]	*MC4R* gene (rs571312, rs2229616 and rs7227255)	**MAF**: N/A. **OR**: N/A **Finding of the study**: *MC4R* rs571312 and rs2229616 are associated with obesity-related traits in Malaysian Malays, but rs7227255 is not. The *MC4R* gene shows low linkage disequilibrium in this population, and its haplotypes do not increase obesity risk.	0.58
5	Ng et al., 2014 [23]	*LEP* G-2548A (rs7799039) *LEPR* Q223R (rs1137101) *TNFα* G-308A (rs1800629) * Except *TNFα* G-308A	**MAF**: *LEP* G-2548A, G = 0.247 (Chinese), 0.336 (Malay); *LEPR* Q223R, A = 0.139 (Chinese), 0.232 (Malay), 0.438 (Indian); and *TNFα* G-308A, A = 0.096 (Chinese), 0.058 (Malay) and 0.062 (Indian). **OR**: *LEP* **G-2548A** male adolescents with AA genotype: 3.64 (95%CI: 1.15–11.54; *p* = 0.025), *LEP* G-2548A 2.10 (overweight/obese): OR, 2.10 (95%CI: 0.98–4.48; *p* = 0.053), (over-fat) Indian subjects: 2.63 (95%CI: 1.14–6.03; *p* = 0.020). **Finding of the study**: *LEP* G-2548A risk allele may be associated with overweight/obese Indian male adolescents in Malaysia.	1.00
6	Fan and Say 2014 [25]	Leptin (*LEP*) gene (G2548A and A19G) Leptin Receptor (*LEPR*) gene (Q223R and K109R)	**MAF**: The *LEP* A19G (0.74), G2548A (0.67) and *LEPR* K109R (061), Q223R (0.79). **OR**: N/A. **Finding of the study**: The *LEP* and *LEPR* SNPs examined in this study may not serve as reliable markers for obesity in this Malaysian population.	0.64
7	Shamsuddin et al., 2018 [37]	G2548A H1328080 A19G	**MAF**: (Case vs. Control): G22548A:(0.32 vs. 0.33), H1328080: (0.25 vs. 0.23), A19G: (0.26 vs. 0.29). **OR**: AAG haplotype of G2548A, H1328080, and A19G: 8.897 (95%CI: 1.59–49.78, *p* = 0.002). **Finding of the study**: Haplotype AAG of G2548A, H1328080, and A19G conferred the significant association with obesity among Malay population in Terengganu.	0.05
8	Mohanraj et al., 2022 [46]	Leptin (*LEP*) gene G2548A Leptin Receptor (*LEPR*) gene Q223R	**MAF**: N/A **OR**: The association of the *LEP* G2548A and *LEPR* Q223R gene variants with BMI (overweight to morbidly obese) were not significant. **Finding of the study**: While leptin (G2548A) and leptin receptor (Q223R) polymorphisms do not have a direct association with BMI or related factors in the population examined, other factors like gender, ethnicity, and psychological state significantly influence plasma leptin levels.	0.37
(b) Polygenic obesity				
**No**	**Author**	**Gene Variants**	**Outcomes (Minor Allele Frequency (MAF)/Odd Ratio (OR)**	**Estimated Power of Study**
1	Yiew et al., 2010 [15]	*PPAR* L162V; *PPAR2* C161T; *UCP1* −3826A/G; *UCP2* 45 bp Ins/Del and −866G/A *UCP3* −55C/T SNPs	**MAF**: *PPAR* L162V (0.006); *PPAR2* C161T (0.36); *UCP1* −3826A/G (0.58); *UCP2* −866G/A (0.12), 45 bp I/D (0.56) and *UCP3* −55C/T (0.34). **OR**: N/A. **Finding of the study**: None were associated with obesity.	0.07
2	Chan et al., 2011 [16]	*PYY* gene (rs1058046) (T)	**MAF**: 0.45 N/A. **OR**: The mutated TT genotype and T allele were both not associated with obesity and the OR for obesity was 0.946 for those with T allele. **Finding of the study**: R72T variant in *PYY* gene was not associated with obesity and most of its related anthropometric measurements.	0.05
3	Lisa et al., 2011 [17]	*CARTPT* rs2239670 (A)	**MAF**: 0.17. **Unadjusted OR**: 0.977 (95%CI: 0.639, 1.492, *p* = 0.913); **Adjusted OR**: 0.809 (95%CI: 0.511, 1.280, *p* = 0.365); Adjusted for age, gender, and ethnicity. **Finding of the study**: *CARTPT* rs2239670 was not a predictor of obesity in the Malaysian subjects of this study.	0.05
4	Apalasamy et al., 2012 [20]	Regions of the *FTO* gene	**MAF**: *FTO* gene polymorphisms ranged from 0.126 to 0.355. **OR**: N/A. **Finding of the study**: No specific haplotype was significantly associated with an increased risk of obesity in Malaysian Malays.	0.54
5	Chey et al., 2013 [21]	*FTO* rs9939609 (T)	**MAF**: 0.199. **Unadjusted OR**: 1.680 (95%CI: 1.036, 2.72, *p* = 0.035). **Adjusted OR**: 1.455 (95%CI: 0.874, 2.42, *p* = 0.149); Adjusted for age, gender, and ethnicity. **Finding of the study**: No link found between this SNP and obesity or related traits, even though the MAF was highest among Malays.	0.12
6	Appalasamy et al., 2014 [24]	*ADIPOQ* (rs3774261 and rs17366568)	**MAF**: rs3774261 (0.46), rs17366568 (0.04). **OR**: 2.15 (95%CI: 1.13–4.09, *p* = 0.026) and 0.87 (95%CI: 0.67–1.13, *p* = 0312), respectively. **Finding of the study**: Only *ADIPOQ* rs17366568 polymorphism was associated with obesity.	0.75
7	Apalasamy et al., 2014 [26]	Insulin-induced gene 2 (*INSIG2*) (rs7566605)	**MAF**: N/A. **OR**: N/A **Finding of the study**: *INSIG2* rs7566605 SNP is not an important variant in predisposing Malaysian Malays to obesity.	0.58
8	Say et al., 2014 [27]	Uncoupling Protein 2 gene (*UCP2*) 45-bp I/D polymorphism	**MAF**: Overall (0.14), Malay (0.17), Chinese (0.12), Indian (0.21). **OR**: I/D genotype (2.02 (95%CI: 1.18, 3.45; *p* = 0.01), I allele (1.81 (95%CI: 1.15, 2.84, *p* = 0.01). **Finding of the study**: *UCP2* 45-bp I/D polymorphism was associated with obesity and overall adiposity (total body fat percentage) among women in this cohort.	0.99
9	Appalasamy et al., 2015 [28]	*RETN* (rs34861192, rs1862513, and rs3219175)	**MAF**: rs1862513 (0.46), rs3219175 (0.14), rs34861192 (0.15). **OR**: 0.86, 1.03, and 0.8, respectively (All OR values were not significant). **Finding of the study**: The haplotypes of the *RETN* gene were not associated with obesity.	0.05
10	Apalasamy et al., 2015 [29]	rs1042714 (Gln27Glu)	**MAF**: N/A. **OR**: N/A. **Finding of the study**: rs1042714 polymorphism may play a key role in the development of obesity-related traits in Malaysian Malays, with gender influencing its impact on these traits.	N/A
11	Chia, et al., 2015 [30]	*PPAR* genes *PPARδ* T294C SNPs *PPARγ2* C161T *PPARα* L162V	**MAF**: Overall: *PPAR α* L162V (0.08), *PPARγ2* C161T (0.22) and *PPARδ* T294C (0.30), respectively. **OR**: No association was found between obesity and *PPARα* L162V, *PPARγ2* C161T, and *PPARδ* T294C SNPs. **Finding of the study**: None of the *PPAR* SNPs were associated with obesity or Metabolic syndrome in the suburban population of Kampar, Malaysia.	0.36
12	Zain et al., 2015 [31]	*NPY* (rs16147 and rs5574)	**MAF**: *NPY* rs16147 T allele: (0.44 vs. 0.38, obese vs. control respectively); *NPY* rs5574 T allele: (0.28 vs. 0.33, obese vs. control respectively). **OR**: rs16147 T allele: 1.46 (95%CI: 1.02–2.07; *p* = 0.036), rs5574 T allele: 0.63 (95%CI: 0.46–0.86; *p* = 0.02). **Finding of the study**: The rs16147 T allele contributed towards an increased risk of obesity, whereas the rs5574 T-allele conferred reduced risk.	0.34
13	Zaharan et al., 2018 [32]	*FABP2* rs1799883 β-3 adrenergic receptor gene *ADRB3* (rs4994) *MC3R* (rs3827103) *GHRL* (rs696217) vit D receptor (rs2228570)	**MAF**: rs1799883 (0.25), rs4994 (0.01), rs3827103 (0.25), rs696217 (0.08) and rs2228570 (0.33) **OR**: N/A. **Finding of the study**: *ADRB3* rs4994 and *MC3R* rs3827103 were associated with % body fat (BF).	0.55
14	Zahri et al., 2016 [33]	Peroxisome proliferator-activated receptor g2 (*PPARγ2*) gene; Pro12Ala polymorphism	**MAF**: Pro12—obese (0.941), Non-obese (0.989). Ala12—obese (0.059), Non-obese (0.011). **Unadjusted OR**: Pro12Ala −5.30 (95%CI: 1.44–19.59, *p* = 0.012). **Adjusted OR**: 5.46 (95%CI: 0.27.0–23.40, *p* = 0.022) (adjusted for age, TG and LDL-C). **Finding of the study**: The Pro12Ala polymorphism in the *PPARγ2* gene predisposes Malay individuals to obesity, and the Ala12 allele may predict changes in lipid metabolism and adipocyte in this population.	1.00
15	Shunmugam et al., 2016 [34]	Angiotensin-converting enzyme (*ACE*) genes α-adrenergic receptor 2A (*ADRA2A*)	**MAF**: Overall = *ADRA2A* rs553668 (0.55), *ACE* I/D (0.56). **Unadjusted OR**: *ACE* II genotype and I allele: 2.15 (95%CI: 1.02–4.52, *p* =0.04) and 1.55 (95%CI: 1.05, 2.28), *p* = 0.03) respectively. **Adjusted OR**: 2.02 (95%CI: 0.87, 4.70, *p* = 0.10) and 1.46 (95%CI: 0.95, 2.26, *p* = 0.09) (adjusted for gender, age, and ethnicity). **Finding of the study**: Subjects with both *ADRA2A* rs553668 GG and *ACE* I/D II genotypes had notably lower WHR compared to other genotype combinations which suggests ACE II genotype may serve as a protective factor against central adiposity risk.	0.52
16	Kok et al., 2017 [35]	Interleukin-4 (*IL4*) intron 3 70 bp Interleukin-1 receptor antagonist (*IL1RA*) intron 2 86 bp repeat	**MAF**: *IL1RA* (0.02) and *IL4* (0.25). **OR**: IL1RA (I/II Genotype): 12.21 (95%CI: (2.54–58.79, *p* = 0.002), II Allele: 5.78 (95%CI: 1.73–19.29, *p* = 0.004) (adjusted for ethnicity). **Finding of the study**: *IL1RA* intron 2 VNTR appears to be a strong genetic marker for overall adiposity status in the studied population.	0.05
17	Rahmadhani et al., 2017 [36]	BsmI (rs1544410) in the intronic region of the *VDR* gene	**MAF**: N/A. **Adjusted OR**: GA genotype: 1.44 (95%CI: 0.77–1.91, *p* = 0.42). AA genotype: 1.21 (95%CI: 0.60–3.46, *p* = 0.40). A allele of the *VDR* BsmI SNP: 1.21 (95%CI: 0.86–1.70, *p* = 0.28). Adjustment made for gender, ethnicity, maternal education and puberty stage. Finding of the study: *VDR* BsmI polymorphism was not associated with obesity.	0.84
18	Mitra et al., 2018 [38]	*FTO* (rs9930506)	**MAF**: 0.365. G allele is the minor allele among the studied ethnic groups, particularly in the Indian compared to Chinese and Malays population (78% vs 49.2%, *p* < 0.001). **Unadjusted OR**: GG vs. AA: 2.65 (95%CI: 1.09–6.45, *p* = 0.032). **Adjusted OR**: 2.87 (95%CI: 1.14–7.19, *p* = 0.025) adjusted for age, sex, physical activity, smoking, and alcohol use. **Finding of the study**: The risk allele (G) of *FTO* rs9930506 was not associated with a higher risk of obesity.	N/A
19	Lek et al., 2018 [39]	*DRD2* Taq1A, Taq1B and Taq1D gene polymorphisms	**MAF**: Taq1A, Taq1B and Taq1D = Chinese (0.37, 0.39, 0.06), Indian (0.29, 0.28, 0.30). **OR**: The *DRD2* Taq1A, Taq1B, and Taq1D genotypes and alleles showed no overall association with BMI, total body fat, or waist-hip ratio classes. However, the Taq1D D2 allele was linked to a 0.55 times lower risk of high central adiposity (WHR) compared to the D1 allele (OR: 0.55 (95%CI: 0.33–0.93, *p* = 0.03). **Finding of the study**: *DRD2* Taq1 gene polymorphisms influence eating behavior and preference, intake frequency, and craving for high-fat foods in Malaysian adults, but their impact on obesity and adiposity remains inconclusive.	0.05
20	Chong et al., 2018 [40]	*IRX3* (rs3751723)	**MAF**: N/A. **OR**: homozygous G/G vs. T/T: 1.72 (95%CI = 1.02–2.91, *p* < 0.05) **Finding of the study**: The G/G genotype was linked to a higher obesity risk in non-fast-food consumers. The G allele increased overweight risk in Malaysian females but was protective in smokers. However, meta-analysis found no significant association between the *IRX3* rs3751723 polymorphism and obesity.	0.97
21	Chong et al., 2018 [41]	Fatty acid synthase (*FASN*) gene (rs2229422, rs2228305, rs2229425, and rs4246445)	**MAF**: rs4246445 (0.363), rs2229422 (0.186), rs2228305 (0.022) and rs2229425 (0.003). **OR**: N/A. **Finding of the study**: The four SNPs were independent to each other, and not all of the haplotypes identified were significantly associated with overweight and obesity in this study.	0.08
22	Mitra et al., 2019 [42]	*ADRB2* (rs1042713)	**MAF**: 0.49 **OR**: No significant association between ADRB2 rs1042713 and obesity (obesity as defined by BMI ≥ 27.5 kg/m^2^) under codominant (AG: 1.26, (95%CI: 0.59–2.71, *p* = 0.548) and GG: 0.94, (95%CI: 0.40–2.23, *p* = 0.884), dominant: 1.14, (95%CI: 0.56–2.33, *p* = 0.725), and recessive: 0.80, (95%CI: 0.40–1.61, *p* = 0.538) models, after adjusting for covariates age, gender, physical activity status, smoking status, and alcohol consumption. **Finding of the study**: No link was found between *ADRB2* rs1042713 and obesity in Malaysian adults; however, it was associated with insulin resistance.	0.47
23	Al-Shajrawi et al., 2020 [43]	*NFKB1* (rs28362491) *HIF1* (rs11549465)	**MAF**: N/A. Genotypes: *HIF-1* (rs11549465); CC: 84% and CT: 16% NFKB1 (rs28362491); Ins/Ins: 25%, Ins/Del: 44.7% and Del/Del: 30.3%. **OR**: N/A **Finding of the study**: A significant association was found between *NFKB1* rs28362491 and obesity (*p* = 0.002). Combination of CC for rs11549465 and Ins/Ins for rs28362491 were significant predictors for obesity, alongside waist circumference and LDL levels in the study population.	0.05
24	Lim et al., 2020 [44]	Dopamine receptor gene variants (*DRD2/ANKK1*) Taq1A (rs1800497), *DRD2* Taq1B (rs1079597) and *DRD2* Taq1D (rs1800498))	**MAF**: Taq1A rs1800497 (0.38), Taq1B rs1079597 (0.39), Taq1D rs1800498 (0.08). **OR**: N/A **Finding of the study**: The *ANKK1/DRD2* Taq1A gene variant may significantly impact emotional eating in Malaysian adults. However, no association was found between *DRD2/ANKK1* variants and obesity.	N/A
25	Tan et al., 2020 [45]	*FTO* (rs9932754, rs9930501 and rs9930506) *ADRB2* (rs1042714 and rs1042713)	**MAF**: rs9930506 (0.37), rs993050l (0.37), rs9932754 (0.37), and rs1042713 (0.13). **OR**: 2.87 (95%CI: 1.14–7.19), 3.03 (95%CI: 1.23–7.49), 3.04 (95%CI: 1.22–7.59) and 1.38 (95%CI: 0.08–23.93), respectively. **Finding of the study**: The highest tertile of polygenic risk score was significantly linked to increased odds of elevated C-reactive protein concentrations, indicating that individuals with a greater number of obesity-related risk alleles tend to have higher CRP levels.	0.85
26	Ching et al., 2023 [47]	Fatty acid desaturase 1 (*FADS1*) gene (rs174547)	**MAF**: N/A **OR**: rs174547 and fiber intake was significant for vegetarians with the TT genotype at tertile 2 fiber intake after adjusting for age, sex, ethnicity, and food groups, OR: 4.71 (95%CI: 1.25–17.74, *p* = 0.022). **Finding of the study**: s174547 SNP in the *FADS1* gene significantly interacts with fiber intake in relation to abdominal obesity among middle-aged Malaysian vegetarians, specifically those with the TT genotype.	0.29

**Table 3:** GO Biological Process and KEGG pathways of genes associated with monogenic obesity (*POMC, MC4R, LEP*, and *LEPR*).

GO Biological Process	Strength	False Discovery Rate (FDR)
Response to melanocyte-stimulating hormone	3.52	0.0013
Leptin-mediated signaling pathway	2.95	0.0056
Regulation of appetite	2.65	0.0130
Regulation of feeding behavior	2.58	0.0114
Bone growth	2.64	0.0133
Regulation of bone remodeling	2.6	0.00029
Response to dietary excess	2.59	0.0144
Regulation of endocrine processes	2.4	0.019
Energy reserve metabolic process	2.47	0.00032
Insulin secretion	2.44	0.0247
Regulation of gluconeogenesis	2.41	0.0257
**KEGG Pathways**		
Adipocytokine signaling pathway	2.34	3.01 × 10 ^−5^
AMPK signaling pathway	2.04	0.0253
Non-alcoholic fatty liver disease	1.95	0.0280
JAK-STAT signaling pathway	1.92	0.0280
Neuroactive ligand-receptor interaction	1.78	2.69 × 10^−5^

## Data Availability

The original contributions presented in the study are included in the article, further inquiries can be directed to the corresponding author.
